# Plasma microRNA-720 may predict prognosis and diagnosis in glioma patients

**DOI:** 10.1042/BSR20201449

**Published:** 2020-07-15

**Authors:** Peng Chen, Guangying Zhang, Qin Zhou, Zhanzhan Li

**Affiliations:** 1 Department of orthopedic, Xiangya Hospital, Central South University, Changsha, Hunan Province 410008, China; 2 Department of Oncology, Xiangya Hospital, Central South University, Changsha, Hunan Province 410008, China

**Keywords:** Diagnosis, Glioma, Micro RNA, Prognosis

## Abstract

We enrolled 122 patients with glioma who received surgery treatment in our hospital from June 2010 to May 2012, and 60 healthy individuals. We found that the plasma miR-720 in the glioma group was significantly higher than that in the healthy control group (3.19 ± 1.26 vs 0.98 ± 0.65, *P*<0.001). The sensitivity and specificity were 71.3% (95%CI: 62.4–79.1%) and 83.3% (71.5–91.7%), respectively. The results indicated that the plasma miR-720 level was associated with tumor grade (*t* = 104.418, *P*<0.001). The advanced tumor tended to have higher miR-720 expression level. No significant association was found between miR-720 and age, sex, tumor size, KPS and tumor position (*P*=0.438, 0.514, 0.518, 0.058, 0.226). The multivariate cox analysis indicated that the high expression of miR-720 (HR = 1.48, 95%CI: 1.12–2.97, *P*=0.023) was independently predictors of adverse prognosis in patients with glioma. The high expression of miR-720 was also associated with recurrence or development in patients with glioma (HR = 1.47, 95%CI: 1.18–3.14, *P*=0.012). Plasma miR-720 has a moderate diagnostic ability in early diagnostic of glioma and may be a potential tumor biomarker. The high plasma miR-720 was related to adverse prognosis in patients with glioma and could be a prognosis predictor of glioma patients.

## Introduction

Glioma is the most common human primary malignant brain tumors, accounting for approximately 60% of all central nervous system rumors. Glioma had a rapid infiltrative growth pattern, which makes complete surgical resection impossible [1]. Currently, patients with glioma still had high mortality and poor 5-year survival rate despite the fact that the diagnosis and treatment, including surgery, radiotherapy and chemotherapy have achieved important development. The poor prognosis is due to the early local invasiveness as well as the lack of effective early diagnosis. The golden standard of glioma diagnosis is histological evaluation [2]. However, its obstacle is still acquiring tissue owing to the special position of glioma. Therefore, it is extremely necessary to explore novel and highly sensitive molecular biomarker with reliable clinical significance.

The microRNAs are groups of non-coding RNAs with 20–22 nucleotides, which negatively regulate the expression of target gene by repressing the translation of target mRNAs [3]. Previous evidences indicated that miRNAs were great significances in the crucial biological process such as cellular proliferation, differentiation and tumorigenesis [4]. The aberrant expression of miRNAs had been identified in many tumors, and its expression profiles were different among different types of tumor. Moreover, circulating miRNAs have been reported to be convenient and non-invasive biomarkers with high stability, indicating great potential [5]. Recently, several studies had explored the feasibility whether the abnormal single miRNAs could be used as potential diagnostic or prognostic biomarkers in glioma such as miR-590, miR-378 and miR-210 [6–9]. However, the sensitivity and specificity were not good for clinical practice. MiR-720 is an oncogene that is dysregulated in many human cancers, and its overexpression contributes to the growth, invasion and or chemotherapy sensitivity of these tumors. Previous studies revealed that miR-720 were significantly up-regulated in tissues, which provides us a hint that miR-720 could be a promising biomarker for early diagnostic and prognosis [10]. Although circulating miR-720 had been found in the plasma of some tumors, and its expression and correlation with clinical features in glioma had not been explored. Therefore, we detected the expression of plasma circulating miR-720 in glioma patients and health controls to assess its role of diagnostic and prognosis prediction. Furthermore, we evaluated the association among clinical data clinic pathological variables, and diagnostic or prognosis value. Our results provided a new evidence that plasma miR-720 may a potential diagnostic and prognosis biomarker with satisfactory sensitivity and specificity in patients with glioma.

## Materials and methods

### Study population

We enrolled 122 patients with glioma who received surgery treatment in our hospital from June 2010 to May 2012, and 60 healthy individuals. Criteria for inclusion and exclusion: all patients were confirmed by postoperative pathology and did not received preoperative chemotherapy or radiotherapy. Patients with other type of tumor and severs immune disease, and inflammation disease were excluded. There were 78 males and 44 females, with a mean age of 48.6 ± 6.01. The mean diameter of tumor was 3.72 ± 1.18. All patients were categorized according to World Health Organization stage of central nervous system tumor (2016) [11]: Stage I: 20, Stage II: 17, Stage III: 35 and Stage IV: 50. The research has been carried out in accordance with the World Medical Association Declaration of Helsinki, and that all subjects provided written informed consent.

### Data collection

We followed up once a month after operation in the first 2 years, and once 3 months in the following 3 years. All patients were followed up by July 2017. The primary outcome was death. The overall survival was from the inception to time of death caused by any accidents, and the disease-free survival was from the inception to disease recurrence or development. The general characteristics were also collected, including sex, age, Karnofsky Performance Status (KPS) score and pathology stage. The present study was approved by the Ethical Committee of Xiangya Hospital.

### Extraction of microRNA

The preoperative and postoperative fasting venous blood was extracted by using a 4 ml anticoagulation tube. The blood samples in the drying test tube were centrifuged for 5 min with a 9-cm centrifuge radius at 3000 r/min, and the plasma was preserved for detection in the refrigerator with −70°C.

According to the instructions of the miRNeasy Serum/Plasma Kit (Qiagen, Hilden, Germany), we extracted the total RNA of cells. About 5 μl extracted RNA the RNase-Free ddH_2_O to 50 mμl was mixed and diluted to 50 μl. The A260 and A280 were measured by quartz microcolorimetric utensil with the control RNase-Free ddH_2_O (1.8–2.1). According to the quantitative results of the A260, the specific amount of cDNA of 12 groups of RNA extraction products were obtained by taking 1 mμ gRNA (reverse transcription of the anti-transcription kit). The sample was completely mixed, experienced brief centrifugation and 37°C incubation for 60 min. The reverse transcription product was preserved at −20°C. We used the primer express 3.0 software to design and synthesis the has-miR-720 primers sequence for UCUCGCUG-GGGCCUCCA according to the mRNA sequence of target gene. Both of target gene probe and the endoginseng gene probe were 5′ end with FAM report fluorescent marker, and 3′ end with TAMRA quenching fluorescence labeling. About 2 μg RNA, internal U6, purpose RNA CD133, and miR - 145 reverse transcription primers were put together at 70°C for 10 min and take out immediately for 2-min ice bath, then add other reagents. The reaction procedure is: 42°C for 60 min, 70°C for 10 min. cDNA products was fast cooled at −80°C for standby application. The real-time quantitative PCR amplification was conducted by using reverse transcription product templates. The reaction condition: 95°C for 3 min with a starting template degeneration; 95°C for 15s, PCR template degeneration during the cycle, then 60°C in 45 s reaction for annealing, experienced for 35 cycles. The relative quantitative expression of miRNA was calculated by relative quantitative method, and the relative expression of gene was analyzed by 2-delta CT method.

### Statistical analysis

We used the SPSS 19.0 to finish all the analyses. The plasma miRNA-720 was expressed as mean ± standard deviation. We compare the differences of miRNa-720 in age (≥45 vs <45), sex (male vs female), grade (I, II, III, IV), KPS (<90 vs ≥90), and tumor position (Frontal lobe, Temporal lobe, Multiple lobes, and other). The receiver operating characteristic (ROC) curve was used to assess the diagnostic ability of mRNA-720 for glioma. The univariate and multivariate Cox regression was used to assess the effect of miRNA-720 on prognosis in patients with glioma. Hazard risk (HR) and relative 95% confidence interval were calculated. We used the log-rank test to compare the overall survival rate and disease-free survival rate between low and high expression of miRNA-720 groups (divided by mean). *P*<0.05 was considered as to be significant.

## Results

### Diagnostic value of plasma miRAN-720 for glioma

**Figure 1:**
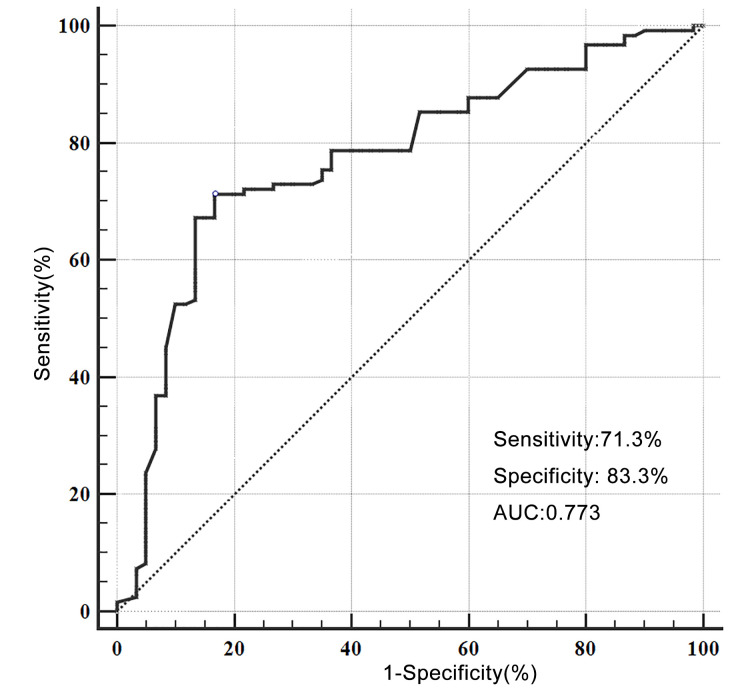
The receiver operating characteristic curve of miR-720 for glioma (AUC = 0.773, sensitivity: 71.3%, specificity: 83.3%, positive predictive value: 89.7%, negative predictive value: 58.8%, cut-off: 3.19)

We detected the expression levels of plasma miRNA-720 in glioma patients and healthy controls. We found that the plasma miR-720 in the glioma group was significantly higher than that in the healthy control group (3.19 ± 1.26 vs 0.98 ± 0.65, *P*<0.001). The Figure 1 presented the diagnostic value of miR-720 between and glioma patients and healthy population. The sensitivity and specificity were 71.3% (95%CI: 62.4–79.1%) and 83.3% (71.5–91.7%) with a cutoff value of 3.19, respectively. The positive likely ratio and negative likely ratio were 4.28 and 0.34, and the positive predictive value: 89.7%, negative predictive value: 58.8%, respectively. The area under the curve was 0.773 (95%CI: 0.706–0.832, *P*<0.001). The results indicated that the diagnostic ability of miR-720 for glioma was moderate.

### Relationship between plasma miRNA-720 and clinical features in glioma patients

The results of relationship between miRNA-720 and clinical features are presented in Table 1. We compare the differences of miRNa-720 in age (≥45 vs <45), sex (male vs female), grade (I, II, III, IV), KPS (<90 vs ≥90), tumor size and tumor position (frontal lobe, temporal lobe, multiple lobes and other). The results indicated that the plasma miR-720 level was associated with tumor grade (*t* = 104.418, *P*<0.001). The higher-grade tumor tended to have higher miR-720 expression level. The miR-720 level was significantly higher in the grade IV than that in stage III, stage II and stage I (*P*<0.05). The stage III was higher than stage II and stage I (*P*<0.05). There was no significant difference between stage I and stage II (*P*>0.05). No significant association were found between miR-720 and age, sex, tumor size, KPS and tumor position (*P*=0.438, 0.514, 0.518, 0.058, 0.226).

**Table 1:** Comparison of clinical parameters and serum levels of miR-720 expression in patients with glioma

Parameters	*N*	miR-720	*t/F*	*P*
Age			−0.778	0.438
≥45	65(53.3%)	3.12 ± 1.16		
<45	57(46.7%)	3.28 ± 1.10		
Sex			0.662	0.514
Male	78(63.9%)	3.15 ± 1.24		
Female	44(36.1%)	3.27 ± 1.17		
Tumor size			-0.649	0.518
>5cm	46(37.7%)	3.28 ± 1.20		
≤5cm	76(62.3%)	3.14 ± 1.19		
WHO grade			104.418	0.000
I	19(15.6%)	1.20 ± 1.10		
II	21(17.2%)	1.37 ± 1.15		
III	35(28.7%)	2.26 ± 1.20		
IV	47 (38.5%)	5.51 ± 1.18		
KPS			-1.910	0.058
<90	50(41.0%)	2.95 ± 1.18		
≥90	72(59.0%)	2.37 ± 1.18		
Tumor position			1.472	0.226
Frontal lobe	33(27.0%)	3.21 ± 0.92		
Temporal lobe	34(27.9%)	3.15 ± 1.08		
Multiple lobes	25(20.5%)	3.34 ± 1.16		
Other	30(24.6%)	3.01 ± 1.43		

### Influences of plasma miRNA-720 on glioma patient’s prognosis

**Figure 2:**
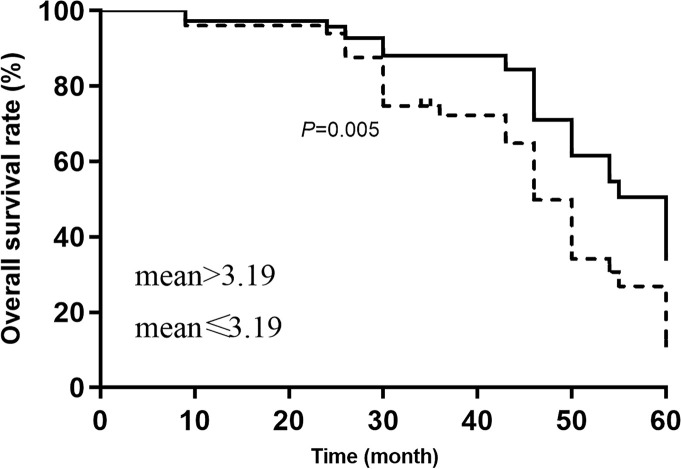
Comparison of overall survival rate between high expression and low expression of miR-720 (mean > 3.19 vs mean ≤ 3.19)

**Figure 3:**
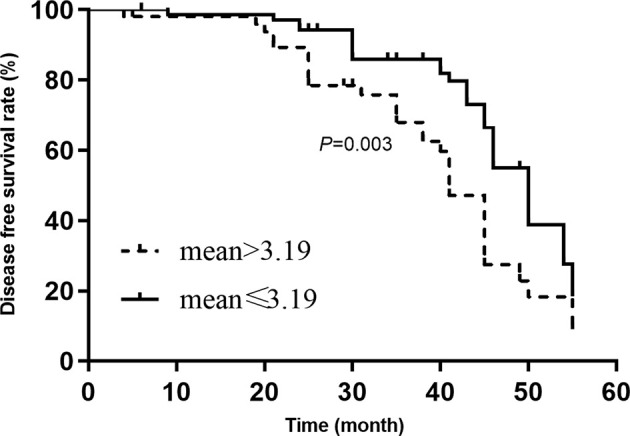
Comparison of disease-free survival rate between high expression and low expression of miR-720 (mean > 3.19 vs mean ≤ 3.19)

According to the mean of miRNA expression, the study population was divided into low expression group and high expression group. The Kaplan–Meier assessed the influences of miR-720 on overall survival rate and disease-free survival rate. The Figure 2 showed that the 5-year survival rate in the high expression group was significantly lower than that in the low expression level group (*P*=0.005). The disease-free survival rate in the high expression group was also lower than that in the low expression group (*P*=0.003, Figure 3). We treated the primary outcomes (death) and recurrence or development as the dependent variables, and age (≥45 vs <45), sex (male vs female), grade (I, II, III, IV), KPS (<90 vs ≥90), tumor size (>5 cm vs ≤5 cm) was considered as to be independent variables. The univariate cox regression indicated that KPS<90, Stage III and IV, high expression of miR-720 was associated with adverse prognosis (overall survival rate and disease-free survival rate) in patients with glioma. The multivariate results indicated that the KPS<90 (HR = 1.23, 95%CI: 1.05–2.34), WHO grade III and IV (HR = 1.17, 95%CI: 1.08–2.59) and high expression of miR-720 (HR = 1.48, 95%CI: 1.12–2.97, *P*=0.023) were independently predictors of adverse prognosis in patients with glioma. The high expression of miR-720 was also associated with recurrence or development in patients with glioma (HR = 1.47, 95%CI: 1.18–3.14, *P*=0.012). The other results were presented in Tables 2 and 3.

**Table 2:** Univariate and multivariate COX regression analyses of the relationships between clinical parameters and overall survivial

Parameters	Category	Univariate	*P*	Multivariate	*P*
Age	≥45 vs <45	1.26(0.85–2.67)	0.396	1.12(0.74–2.15)	0.645
Sex	Female vs Male	1.12(0.65–1.87)	0.498	1.02(0.53–1.62)	0.501
Tumor size	>5 cm vs ≤5 cm	2.32(0.92–3.15)	0.642	2.15(0.86–3.05)	0.573
KPS	<90 vs ≥90	1.67(1.18–2.69)	0.014	1.23(1.05–2.34)	0.035
WHO grade	III IV vs I II	1.36(1.23–2.68)	0.025	1.17(1.08–2.59)	0.008
miR-720	High vs Low	1.59(1.15–3.64)	0.002	1.48(1.12–2.97)	0.023

**Table 3:** Univariate and Multivariate COX regression analyses of the relationships between clinical parameters and disease-free survival

Parameters	Category	Univariate	*P*	Multivariate	*P*
Age	≥45 vs <45	1.23(0.72–2.46)	0.368	1.46(0.92–2.68)	0.427
Sex	Female vs Male	0.73(0.85–1.64)	0.257	0.85(0.74–2.04)	0.369
Tumor size	>5 cm vs ≤5 cm	1.25(0.69–3.15)	0.842	1.34(0.71–2.96)	0.796
KPS	<90 vs ≥90	1.23(1.16–2.68)	0.016	1.14(1.02–3.46)	0.028
WHO grade	III IV vs I II	1.16(1.09–2.67)	0.032	1.09(1.07–2.56)	0.041
miR-720	High (>3.19) vs Low (≤3.19)	1.54(1.29–3.04)	0.004	1.47(1.18–3.14)	0.012

## Discussion

Our study found that plasma miR-720 was associated with glioma pathology grade, and miR-720 may be a predictor of prognosis in patients with glioma. The high expression of miR-720 means advanced grade tumor and higher risk of adverse outcomes. The miR-720 could be a moderate diagnostic biomarker of glioma, with the sensitivity of 71.3% and specificity of 83.3%. Our results provided some support for clinical diagnostic and treatment for glioma.

As a noncoding RAN, miRNA was suggested to be involved in the proliferation, invasion and apoptosis of tumor cells [12]. These function exerted effects through the regulation of 3′ region of target mRNA to regulate the expression of target gene [13]. It was reported that the abnormal expression of miRNAs was associated with development of glioma [14]. The miR-92b can inhibit the proliferation, migration and invasion of glioma cell by regulating its target gene hosphatase and tensin homolog [15]. miR-506 can also inhibit the proliferation of glioma through targeting IGF2BP1 gene [16]. These results indicated that the miRNAs played an important role in the development of tumor. Glioma is a malignant tumor of central nervous system. The prognosis of patients was still poor although the combination application of chemotherapy resection and postoperative glioma. The survival of patients was usually 14 months [17]. The adverse prognosis of patients with glioma was associated with malignant biological behavior of tumor cells, including proliferation of brain tissue surrounding the invasion and chemotherapy drug resistance [18–20]. It was acknowledged that the excessive proliferation of tumor cells was an important factor causing recurrence of glioma after operation [21–23]. It has been becoming an important issue how to effectively inhibit the excessive proliferation of glioma cells as well as prolonging the survival of patients and improving the quality of life. Our study reported that the plasma expression of miR-720 was significantly in the patients’ group than that in the healthy control group and was positively associated with tumor differentiation. The 5-year survival rate of high expression group was obviously lower than the low expression group. Our results indicated that miR-720 was involved in the development of glioma and was a potential biomarker of prognosis.

Previous studies reported that miR-720 have abnormal expression in the several tumors. It was reported that the high expression of miR-720 was found in the colorectal carcinoma tissue. Moreover, the high expression of miR-720 was related to tumor size, tumor stage, lymph node metastasis and 5-year survival rate, and the expression of miR-720 decreased after operation [24]. The plasma expression level of miR-720 was also a diagnostic index of bone myeloma. It was found that the expression of miR-720 was associated with breast cancer. The expression level of breast cancer was higher than that in the precancerous lesions, and was involved in the development, lymph node metastasis, survival status and prognosis. The present study only reported that the high expression of miR-720 markedly enhanced cell proliferation in the glioma cells [25,26]. The results about the relationship between miR-720 and long-term prognosis are few. Our study provided important supports of clinical practice. We assumed that the miR-720 may participate in the early stage of tumor. Inhibiting the expression of miR-720 could interfere tumor occurrences. Based on the analysis of the theory of transport, inhibiting the expression of miR-720 can improve the prognosis of patients with glioma and improve the survival time. Recently, Huynh and Krishnan reported that inhibiting the expression of mir-182 in the body can significantly inhibit the invasion and metastasis of many malignant tumor cells such as liver cancer [27,28]. If these results in the treatment of patients with glioma was further confirmed that miR-720 can be used as a new treatment target to improve the level of the treatment of gliomas, reducing mortality and improving prognosis of patients.

Our study has several limitations. First, if this miRNA can be detected other cancer, it means it is not specific to glioma and the potential utility in diagnosis will be compromised, leading to false detection or else. Second, the sample size is small, and we calculate the overall survival instead of cancer-specific survival for fully using the all data. Third, we did not explore the molecular mechanism. Further research was required.

In conclusion, plasma miR-720 has a moderate diagnostic value in early diagnostic of glioma and may be potential tumor biomarker. The high plasma miR-720 was related to adverse prognosis in patients with glioma and could be a prognosis predictor of glioma patients. The specific biomechanics still need further research.

## Abbreviations

CI: confidence interval
DNA: deoxyribonucleic acid
HR: hazard risk
KPS: Karnofsky Performance Status
RNA: ribose nucleic acid
ROC: receiver operating characteristics

## References

[1] Li Z., Zhou Q., Li Y., Yan S., Fu J., Huang X. et al. (2017) Mean cerebral blood volume is an effective diagnostic index of recurrent and radiation injury in glioma patients: A meta-analysis of diagnostic test. Oncotarget 8, 15642–15650, 10.18632/oncotarget.14922 28152505 PMC5362512

[2] Miller J.J. and Wen P.Y. (2016) Emerging targeted therapies for glioma. Expert Opin. Emerg. Drugs 21, 441–452 10.1080/14728214.2016.1257609 27809598

[3] Small E.M. and Olson E.N. (2011) Pervasive roles of microRNAs in cardiovascular biology. Nature 469, 336–342 10.1038/nature09783 21248840 PMC3073349

[4] Jun F., Hong J., Liu Q., Guo Y., Liao Y., Huang J. et al. (2017) Epithelial membrane protein 3 regulates TGF-beta signaling activation in CD44-high glioblastoma. Oncotarget 8, 14343–14358 10.18632/oncotarget.11102 27527869 PMC5362410

[5] Kalkan R. and Atli E.I. (2016) The Impacts of miRNAs in Glioblastoma Progression. Crit. Rev. Eukaryot. Gene Expr. 26, 137–142 10.1615/CritRevEukaryotGeneExpr.2016015964 27480776

[6] Li Z.Z., Shen L.F., Li Y.Y., Chen P. and Chen L.Z. (2016) Clinical utility of microRNA-378 as early diagnostic biomarker of human cancers: a meta-analysis of diagnostic test. Oncotarget 7, 58569–58578 10.18632/oncotarget.10707 27448977 PMC5295453

[7] Wang H., Wang T., Shi W., Liu Y., Chen L. and Li Z. (2017) Comprehensive analysis on diagnostic value of circulating miRNAs for patients with ovarian cancer. Oncotarget 8, 66620–66628 10.18632/oncotarget.18129 29029542 PMC5630442

[8] Redova M., Svoboda M. and Slaby O. (2011) MicroRNAs and their target gene networks in renal cell carcinoma. Biochem. Biophys. Res. Commun. 405, 153–156 10.1016/j.bbrc.2011.01.019 21232526

[9] Liu Q., Gao Q., Zhang Y., Li Z. and Mei X. (2017) MicroRNA-590 promotes pathogenic Th17 cell differentiation through targeting Tob1 and is associated with multiple sclerosis. Biochem. Biophys. Res. Commun. 493, 901–908 10.1016/j.bbrc.2017.09.123 28947212

[10] Li C.S. (2013) Relative expressions of serum mir-128 and mir-720 in glioma patients and their clinical significance. Lab. Immun. Clin. Med. 4 242–245

[11] Louis D.N., Perry A., Reifenberger G., von Deimling A., Figarella-Branger D., Cavenee W.K. et al. (2016) The 2016 World Health Organization Classification of Tumors of the Central Nervous System: a summary. Acta Neuropathol. 131, 803–820 10.1007/s00401-016-1545-1 27157931

[12] Chen X., Ba Y., Ma L., Cai X., Yin Y., Wang K. et al. (2008) Characterization of microRNAs in serum: a novel class of biomarkers for diagnosis of cancer and other diseases. Cell Res. 18, 997–1006 10.1038/cr.2008.282 18766170

[13] Calin G.A. and Croce C.M. (2006) MicroRNA signatures in human cancers. Nat. Rev. Cancer 6, 857–866 10.1038/nrc1997 17060945

[14] Sasaki A., Udaka Y., Tsunoda Y., Yamamoto G., Tsuji M., Oyamada H. et al. (2012) Analysis of p53 and miRNA expression after irradiation of glioblastoma cell lines. Anticancer Res. 32, 4709–4713 23155233

[15] Song H., Zhang Y., Liu N., Wan C., Zhang D., Zhao S. et al. (2016) miR-92b regulates glioma cells proliferation, migration, invasion, and apoptosis via PTEN/Akt signaling pathway. J. Physiol. Biochem. 72, 201–211 10.1007/s13105-016-0470-z 26893028

[16] Peng T., Zhou L., Zuo L. and Luan Y. (2016) MiR-506 functions as a tumor suppressor in glioma by targeting STAT3. Oncol. Rep. 35, 1057–1064 10.3892/or.2015.4406 26554866

[17] Sasaki A., Udaka Y., Tsunoda Y., Yamamoto G., Tsuji M., Oyamada H. et al. (2012) Analysis of p53 and miRNA expression after irradiation of glioblastoma cell lines. Anticancer Res. 32, 4709–4713 23155233

[18] Wang L., Qin H., Li L., Feng F., Ji P., Zhang J. et al. (2013) Forkhead-box A1 transcription factor is a novel adverse prognosis marker in human glioma. J. Clin. Neurosci. 20, 654–658 10.1016/j.jocn.2012.03.055 23510544

[19] Xie T., Liu P., Chen L., Chen Z., Luo Y., Chen X. et al. (2015) MicroRNA-15a down-regulation is associated with adverse prognosis in human glioma. Clin. Transl. Oncol. 17, 504–510 10.1007/s12094-014-1265-8 25575767

[20] Amoureux M.C., Coulibaly B., Chinot O., Loundou A., Metellus P., Rougon G. et al. (2010) Polysialic acid neural cell adhesion molecule (PSA-NCAM) is an adverse prognosis factor in glioblastoma, and regulates olig2 expression in glioma cell lines. BMC Cancer 10, 91 10.1186/1471-2407-10-91 20219118 PMC2854115

[21] Ratel D., van der Sanden B. and Wion D. (2016) Glioma resection and tumor recurrence: back to Semmelweis. Neuro. Oncol. 18, 1688–1689 10.1093/neuonc/now201 27765836 PMC5744249

[22] Chen W., Yu Q., Chen B., Lu X. and Li Q. (2016) The prognostic value of a seven-microRNA classifier as a novel biomarker for the prediction and detection of recurrence in glioma patients. Oncotarget 7, 53392–53413 10.18632/oncotarget.10534 27438144 PMC5288195

[23] Zhou X., Liao X., Zhang B., He H., Shui Y., Xu W. et al. (2016) Recurrence patterns in patients with high-grade glioma following temozolomide-based chemoradiotherapy. Mol. Clin. Oncol. 5, 289–294 10.3892/mco.2016.936 27446566 PMC4950878

[24] Wang X., Kuang Y., Shen X., Zhou H., Chen Y., Han Y. et al. (2015) Evaluation of miR-720 prognostic significance in patients with colorectal cancer. Tumour Biol. 36, 719–727 10.1007/s13277-014-2697-z 25286763

[25] Li L.Z., Zhang C.Z., Liu L.L., Yi C., Lu S.X., Zhou X. et al. (2014) miR-720 inhibits tumor invasion and migration in breast cancer by targeting TWIST1. Carcinogenesis 35, 469–478 10.1093/carcin/bgt330 24085799

[26] Das S.G., Romagnoli M., Mineva N.D., Barille-Nion S., Jezequel P., Campone M. et al. (2016) miR-720 is a downstream target of an ADAM8-induced ERK signaling cascade that promotes the migratory and invasive phenotype of triple-negative breast cancer cells. Breast Cancer Res. 18, 40 10.1186/s13058-016-0699-z 27039296 PMC4818899

[27] Huynh C., Segura M.F., Gaziel-Sovran A., Menendez S., Darvishian F., Chiriboga L. et al. (2011) Efficient in vivo microRNA targeting of liver metastasis. Oncogene 30, 1481–1488 10.1038/onc.2010.523 21102518

[28] Krishnan K., Steptoe A.L., Martin H.C., Wani S., Nones K., Waddell N. et al. (2013) MicroRNA-182-5p targets a network of genes involved in DNA repair. RNA 19, 230–242 10.1261/rna.034926.112 23249749 PMC3543090

